# Exploring the triggers of premature and early menopause in India: a comprehensive analysis based on National Family Health Survey, 2019–2021

**DOI:** 10.1038/s41598-024-53536-9

**Published:** 2024-02-06

**Authors:** Sampurna Kundu, Sanghmitra Sheel Acharya

**Affiliations:** Centre of Social Medicine and Community Health, School of Social Sciences, Jawaharlal Nehru University, Delhi, 110067 India

**Keywords:** Risk factors, Health policy

## Abstract

Due to ovarian insufficiency, some women attain menopause at an early age due to lifestyle factors and hormonal imbalances. Menopause occurring before the age of 40 is premature and between 40 and 44 years age is early, since the natural age of menopause lies between 45 and 50. The study estimated the prevalence of both premature and early menopause, and examined the potential associated factors that could trigger its occurrence in India. The National Family Health Survey, conducted during 2019–2021, was used to fulfil the study objective. The study sample was divided into two parts, with age group 15–39 and 40–44 for estimating premature and early menopause, respectively. Cox-proportional hazard model was used for the multivariate analysis. The estimated prevalence of premature menopause is 2.2% and early menopause is 16.2%. Lower educational level, poor economic condition, smoking, fried food consumption, early age at menarche are some of the significant explanatory factors. In India, both the proportion and the absolute number of post-menopausal women are growing, therefore it is critical to revamp public reproductive healthcare facilities to include menopausal health segment in women's health as well. Future detailed micro-studies would help in better understanding of the premature or early menopausal cases.

## Introduction

The event of ovarian failure is termed as menopause. Majority women normally attains menopause in the age bracket of 45 and 55^1^. According to the World Health Organization (WHO), natural menopause is defined as “permanent cessation of menstruation resulting from the loss of ovarian follicular activity”, which is normally recognized after a year-long consecutive amenorrhea^2^. This is an essential physical and hormonal event in a healthy woman’s reproductive cycle. With increasing age, the ovarian function depletes and decreases its production of oestrogen and progesterone hormones, and thus the gradual decline in fecundity^3,4^. So basically, menopause is the transition of a woman’s life from reproductive phase to a non-reproductive phase, which has biological, emotional, sociocultural significance^5^.

The age distribution of menopause is like a Gaussian curve ranging from age 40 to 54, but the general clustering is around 45–55^6^. Some women, due to ovarian insufficiency attains menopause at an early age due to lifestyle factors and hormonal imbalances. Menopause occurring before the age of 40 is premature and between 40 and 44 years age is early, since the natural age of menopause lies between 45 and 50. The cessation of menses is marked by amenorrhea, rise in gonadotrophin levels and oestrogen deficiency^7,8^.

According to a PAN India study by Ahuja (2016), there is a strong association between early onset of menopause and various factors such as illiteracy, poor socio-economic background, underweight, parity, and age at pregnancy^9^. Studies have shown that age at menarche, breastfeeding of previous child, age at first pregnancy, plays a very important role in determining the onset of menopause^10^. There are also effects of nulliparity, usage of oral contraceptive pills, having a live birth or not, on the onset of natural menopause^11,12^. Menopausal age is associated with a number of factors with includes smoking^13,14^, level of education^15^, working status^16^, abortion^17^, body mass index^16^ and food habits^18^. Smoking specifically have shown adverse impacts on reproductive health and heavy smokers were observed to reach menopause earlier^19,20^, thus making it important to study its association. Tobacco consumption has anti-estrogenic impacts on the female body which can lead to estrogen function resistance^20^. There has not been any consistent association between the onset of menopause and the above stated factors.

In the coming decades, both the proportion and total number of Indian women aged 45 and beyond are expected to rise sharply. In India, there were around 96 million women who were 45 years of age or older as of the 2011 census, and this figure is projected to rise to 401 million by 2026^21^. Women in India could, on average, spend 30 years in the postmenopausal stage of life because the average life expectancy at age 45 is 30 years. The post-menopausal population may provide significant problems to the provision of public healthcare in the future due to the health concerns associated with these years, including hypertension, heart disease, osteoporosis, and a deterioration in overall quality of life^22,23^. Increasing urbanization and changing lifestyle pose greater challenges to public health. Identifying factors associated with early menopause are necessary. The age at menopause is associated with the risk of several chronic diseases such as cardiovascular diseases, breast and endometrial cancers, and osteoporosis^24–27^. Although this issue is ignored in India, premature or early menopause has numerous short- and long-term negative health consequences. Health problems related to early onset menopause are not well known in India because the determinants and prevalence of premature menopause are not well documented, and thus comes our major research question regarding the same.

The hypothesis is, to test.

### H_0_

There is no significant effect of socio-economic, demographic, medical and lifestyle behaviour on premature and early onset of menopause against

### H_1_

There is a significant effect of socio-economic, demographic, medical and lifestyle behaviour on premature and early onset of menopause.

Thus, the study aims to estimate the prevalence of both premature and early menopause, and examine the potential associated factors in India.

## Data and methods

### Data source

The study utilizes the latest round of National Family Health Survey, conducted during 2019–2021. The first (1993–1993), second (1998–1999), third (2005–2006), fourth (2015–2016) and fifth (2019–2021) round of National Family Health Survey (NFHS) for analysing the overall trend and the recent round for the rest of the study. The National family health survey (NFHS) is a large-scale, multi-round survey conducted in representative sample of household throughout India. The NFHS is a collaborative project of the International Institute for Population Sciences (IIPS), Mumbai, India; ORC Macro, Calverton, Maryland, USA and the East–West center, Honolulu, Hawaii, USA. The Ministry of Health and Family Welfare (MoHFW), Government of India, designated IIPS as nodal agency, responsible for providing coordination and technical guidance for the NFHS. The fifth round of NFHS was conducted in 2019–2021, with the interruption of Covid-19 pandemic in between. The survey was carried out for 707 districts, as of 31st March 2017. Due to the pandemic lockdown, the fieldwork was in two phases- Phase I covered 17 states and union territories from 17th June 2019 to 30th January 2020; and Phase II covered 11 states and union territories from 2nd January 2020 to 30th April 2021. There were 17 field agencies who collected information from 636,699 households, 724,115 women and 101,839 men^28^.

The pregnant women and lactating mothers during the time of survey are excluded from the analysis. Women who had undergone hysterectomy, that is, having surgical menopause are also excluded. Two separate datasets are created for the analysis and the sample sizes for analysis of premature menopause is 429,446 (ages 15–39), and early menopause is 79,643 (ages 40–44).

### Variable description

#### Outcome

The women who had their last menstrual cycle one or more year ago is considered to be menopausal women, and those who had their last menstrual cycle beyond 12 months below the age of 40, is identified as **prematurely menopausal women**, while menopause between 40 and less than 45 years as **early menopause**. Both are dichotomized as ‘1’ and ‘0’.

### Predictors

#### Socio-economic and demographic

The socio-economic and demographic factors considered in the study to observe the patterns of premature and early menopause across the population subgroups. The educational level of women is categorized as no education, primary, secondary and higher education. Caste is grouped into four categories: scheduled caste (SC), scheduled tribe (ST), other backward class (OBC), and others. Religion is categorized as Hindu, Muslims and Others (including Sikh, Buddhist/Neo-Buddhist, Jain, Jewish, Parsi/Zoroastrian, no religion, and other). Place of residence is given as rural and urban in the survey. In the survey, a household wealth index was calculated by combining household amenities, assets, and durables and categorizing households in a range from poorest to richest, corresponding to wealth quintiles from lowest to highest. The working status of the woman are taken as employed and not employed. Marital status is categorized as- never married, currently married, and widowed/divorced/separated. The states and union territories are recoded according to regions as- North (Chandigarh, Delhi, Haryana, Himachal Pradesh, Jammu and Kashmir, Punjab, Rajasthan); North East (Assam, Arunachal Pradesh, Manipur, Meghalaya, Mizoram, Nagaland and Tripura); Central (Chhattisgarh, Madhya Pradesh, Uttarakhand Uttar Pradesh); East (Bihar, Jharkhand, Odisha, West Bengal); West (Dadra and Nagar Haveli and Daman and Diu, Goa, Gujarat, Maharashtra); South (Andhra Pradesh, Karnataka, Kerala, Puducherry, Tamil Nadu, Telangana, Andaman and Nicobar Islands, Lakshadweep)^29^.

#### Lifestyle behavior

The lifestyle behavior were considered for observing the effect of it on the occurrence of premature and early menopause. Consumption of tobacco is considered from the survey as smoking categorized as no and yes. Regular drinking of alcohol is also dichotomized as no and yes. Unhealthy diet pattern is indicated by regular consumption of fried foods and aerated drinks, again grouped as no and yes.

#### Reproductive

The biological or reproductive factors were considered to observe the plausible determining factors of premature and early menopause. The age at menarche is the age of onset of menstrual cycle, and is grouped as ‘12 or less’, ‘13–15’, and ‘more than 15’. Women with no children ever born, that is, with zero parity were considered as nulliparous and the variable is dichotomized. Age at first birth provided details about the respondents' ages at the time of the birth of their first child. These categories were ‘below 18 years’, ‘18–24 years’, and ‘25 + years’ for this variable. The usage of contraceptive methods to delay or prevent pregnancy was a question that respondents were asked. Injectables, pills, and emergency contraception were all regarded in this study as hormonal contraceptives. Menstrual hygiene was categorized as—hygienic methods (sanitary napkins, locally prepared napkins, tampons, menstrual cup), unhygienic methods (cloths, nothing, others) and both. Termination of pregnancy was dichotomized as yes or no.

#### Anthropometric and Bio-chemical

The respondent's measured mass (weight) and height at the time of the interview were used to calculate body mass index (BMI). The SECA 874 U digital scale was used to measure weight, while the SECA 213 stadiometer was used to measure height. Underweight (18.5 kg/m2), normal (18.5–24.9 kg/m2), and obese/overweight (25 kg/m2) were the outcomes of dividing body weight by square of body height, or BMI. During the survey, blood samples from the respondents were taken for anaemia testing. In this study, haemoglobin levels below 12 g/dL were deemed anaemic, and those above 12 g/dL were deemed not to be anaemic. A finger-stick blood sample was used to measure random blood glucose using an Accu-Chek Performa glucometer and glucose test strips. Diabetics were defined as those with a random blood glucose level of 200 mg/dl or higher.

### Statistical methods

The study variables are described using descriptive statistics at first. Bivariate analysis including cross-tabulation and chi-square tests are done to observe significant associations between premature/early menopause and the correlates.

Estimates of the hazard ratio (HR) for the explanatory variables that are expected to influence menopausal state are obtained using Cox proportional hazard regression models. In the survival analysis, the median age (median survival time) was the age at which 50% of the women were anticipated to still be in their reproductive years. A survival technique is chosen over alternative models, such as logit analysis, because it appropriately handles censored observations, which are a feature of this data set, and takes into consideration the duration from menarche to the event of interest, that is, menopause. The assumption of proportional hazards is tested to eliminate bias in the estimates of HRs by looking at plots of the Schoenfeld residuals and by using time-dependent variables. When computing the Cox partial likelihood using the Efron technique^30^, modifications are performed to account for linked observations (several women reaching menopause at the same age).

Cox-proportionate hazard model will be as follows:

$$h\left( {t|X} \right) = h_{0} \left( t \right){\text{exp}}\left( {\beta \cdot X} \right)$$

where

*t*_*i*_ represents the survival time (premature or early menopause age) for the ith individual.

*h(t)* is the hazard function determined by a set of covariates.

*X*_*i*_ is the vector of covariates for the i^th^ individual.

*β* is the vector of model coefficients that measure the impact of covariates.

The term *h(0)* is called the baseline hazard, that corresponds to the value of the hazard if all the x_i_’s are equal to zero.

The likelihood function of the model is given by

$$L\left( \beta \right) = \mathop \prod \limits_{i = 1}^{n} \left[ {\frac{{e^{{\left( {\beta_{i} \cdot X_{i} } \right)}} }}{{\mathop \sum \nolimits_{{j \in R\left( {t_{i} } \right)}} e^{{\left( {\beta_{j} \cdot X_{j} } \right)}} }}} \right]^{{C_{i} }}$$

where

*n* is the sampled women.

*R(t*_*i*_*)* is set of women who are at risk of premature or early menopause at time *t*_*i*_* ,* for the i^th^ individual.

*C*_*i*_ indicates censored observations for the i^th^ individual.

With respect to the parameter vector *β*, the likelihood function aims to maximise the probability of observing the data.

The model coefficients are usually estimated by partial likelihood estimation techniques, which take into account just those who witness the event or are censored at the time *t*_*i*_ .

The exponential of the associated coefficient represents the hazard ratio (HR) for a predictor variable:

$$HR = e^{{\beta_{j} }}$$

Hazard ratios are interpretated as-

HR = 1: No effect.

HR < 1: Reduction in the hazard.

HR > 1: Increase in Hazard.

A total of four models were fitted with the covariates taken in the following manner:

*Model 1* Socio-economic and demographic factors.

*Model 2* Socio-economic and demographic factors + Lifestyle behaviour.

*Model 3* Socio-economic and demographic factors + Lifestyle behaviour + Reproductive factors.

*Model 4* Socio-economic and demographic factors + Lifestyle behaviour + Reproductive factors + Anthropometric and Bio-chemical factors.

### Ethics approval

The analysis is based on secondary data available in public domain for research; thus, no approval was required from any institutional review board (IRB). The survey agencies had conducted the field work with prior consent from the respondents. The NFHS survey was conducted in accordance with the relevant ethical guidelines and regulations.

## Results

### Sample characteristics

The sample of age 15–39 consists of 2.23% of premature menopausal women. Around 34% of the women resided in urban areas and 66% in rural areas. About 15.15% of the sample women did not have schooling/education. Majority of the sample belonged to Hindu religion (81.17%) and 26% belonged to scheduled caste or scheduled tribe. Around 37% women belong to poor wealth index. About 57.7% of the samples were currently married, 39.5% never married and 2.78% were widowed/divorced/separated. The age at menarche is 13 to 15 years for 79% of these women. Around 48% of the sample is nulliparous. The age at first birth is 18 to 24 years for 66% of the women. There are 8.8% and 10.3% women who use hormonal contraceptives and had terminated pregnancies, respectively. According to the body mass index of the sample, 21.7% are underweight, 15.3% are overweight and 5.2% are obese. There are 53.2% women in the age-group 15–39 who are anemic and 0.8% diabetic (Table 1).

**Table 1:** Percentage distribution of the sample by the study variables for the sample.

	Aged 15–39		Aged 40–44	
	*n*	%	*n*	%
Premature Menopause				
No	4,19,871	97.77		
Yes	9575	2.23		
Early Menopause				
No			66,740	83.8
Yes			12,903	16.2
Current age				
15–19	1,14,462	26.65		
20–24	73,360	17.08		
25–29	70,847	16.5		
30–34	80,145	18.66		
35–39	90,632	21.1		
40			24,095	30.25
41			12,216	15.34
42			16,783	21.07
43			13,754	17.27
44			12,796	16.07
Residence				
Urban	1,45,467	33.87	28,013	35.17
Rural	2,83,979	66.13	51,630	64.83
Education				
No education	65,045	15.15	31,880	40.03
Primary	43,866	10.21	12,092	15.18
Secondary	2,40,817	56.08	28,842	36.21
Higher	79,718	18.56	6829	8.58
Employment				
Not employed	48,484	75.1	7594	64.13
Employed	16,074	24.9	4249	35.87
Wealth index				
Poorest	74,532	17.36	13,166	16.53
Poorer	84,830	19.75	15,216	19.11
Middle	89,070	20.74	16,249	20.4
Richer	91,896	21.4	17,006	21.35
Richest	89,118	20.75	18,006	22.61
Caste				
SC	93,646	21.81	16,061	20.17
ST	39,408	9.18	6990	8.78
OBC	1,84,841	43.04	34,587	43.43
Others	1,11,551	25.98	22,005	27.63
Religion				
Hindu	3,48,561	81.17	65,636	82.41
Muslim	58,433	13.61	9402	11.81
Others	22,452	5.23	4605	5.78
Marital status				
Never married	1,69,588	39.49	1010	1.27
Currently married	247,919	57.73	71,663	89.98
Widowed/divorced/separated	11,939	2.78	6970	8.75
Regions				
Northern	77,900	18.14	15,917	19.99
Western	144,495	33.65	22,952	28.82
Southern	72,503	16.88	13,962	17.53
Eastern	4215	0.98	793	1.00
Central	40,717	9.48	7791	9.78
North-Eastern	89,616	20.87	18,229	22.89
Smoking				
No	4,26,223	99.25	78,190	98.18
Yes	3223	0.75	1453	1.82
Drinking				
No	4,26,841	99.39	78,682	98.79
Yes	2605	0.61	961	1.21
Regular consumption of fried foods and aerated drinks				
No	2,23,609	52.07	44,009	55.26
Yes	2,05,837	47.93	35,634	44.74
Age at Menarche				
12 or less	32,497	17.17		
13–15	1,49,363	78.93		
More than 15	7371	3.9		
Nulliparity				
No	2,24,481	52.27	76,578	96.15
Yes	2,04,965	47.73	3065	3.85
Menstrual hygiene				
Hygienic methods	99,120	52.45		
Unhygienic methods	37,902	20.06		
Both	51,948	27.49		
Age at first birth				
Below 18	49,290	22.71	16,651	21.76
18–24	1,43,887	66.3	47,735	62.39
25+	23,846	10.99	12,120	15.84
Hormonal contraceptive use				
No	3,91,326	91.12	69,570	87.35
Yes	38,120	8.88	10,073	12.65
Had terminated pregnancy				
No	3,85,104	89.67	66,734	83.79
Yes	44,342	10.33	12,909	16.21
BMI category				
Underweight	89,902	21.77	7146	9.25
Normal weight	2,38,138	57.66	41,509	53.75
Overweight	63,371	15.34	20,130	26.06
Obese	21,616	5.23	8446	10.94
Anemic				
No	2,01,075	46.82	37,021	46.48
Yes	2,28,371	53.18	42,622	53.52
Glucose level				
Non-diabetic	4,12,942	99.22	74,465	96.69
Diabetic	3238	0.78	2549	3.31
N	4,16,180		77,014	

Coming to the sample of 40 to 44 aged women, 16.2% of them attained early menopause. The socio-economic and demographic variable distribution are almost similar to the former sample, however here 40% women had no schooling/education. About 89.9% of the samples were currently married, 1.27% never married and 8.75% were widowed/divorced/separated. Around 3.8% of the sample is nulliparous. The age at first birth is 18 to 24 years for 62.4% of the women. There are 12.6% and 16.2% women who use hormonal contraceptives and had terminated pregnancies, respectively. According to the body mass index of the sample, 9.3% are underweight, 26.1% are overweight and 10.9% are obese. There are 3.3% women in the age-group 15–39 who are diabetic (Table 1).

### Trends and patterns of premature and early menopause

**Figure 1:**
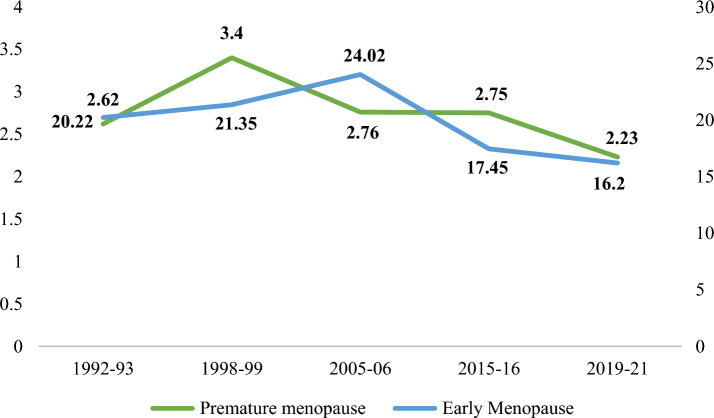
Trends in prevalence of premature and early menopause from 1992 to 2021.

The prevalence of premature and early menopause has decreased gradually over time. Premature menopause was highest during 1998–1999, 3.4% and reduced to 2.76% in 2005–2006 and remained almost constant in the next five years also, and further reduced to 2.23% in recent years. Early menopause was around 21% during the 90 s, increased to 24% in 2005–06, then reduced to 17.5% in the next five years, to 16% in recent years (Fig. 1).

### Correlates of premature and early menopause

The bivariate analysis results for premature menopause sample aged 15–39 are displayed in Table 2. Premature menopause is highest in the age-group 35–39, with 6.1%. Women residing in rural areas (2.6%), has no education (6.1%), employed (2.7%), belonging to poor wealth quintile (2.8%), and other backward class (2.5%), has significantly higher percentage of experiencing premature menopause. Marital status showed and significant association with premature menopause, with around 4.1% prevalence among those who were widowed/divorced/separated. Regional prevalences showed that Northern (2.65%) and Western (2.63%) regions had relatively higher proportions of premature menopause.

**Table 2:** Cross-tabulations and chi-square tests of premature menopause with the possible correlates (age group 15–39).

	Premature Menopause (%)	χ^2^ value	DF	*p*-value
Age-group				
15–19	0.42	9.00E+03	4	< 0.001
20–24	0.55			
25–29	1.07			
30–34	2.96			
35–39	6.13			
Residence				
Urban	1.5	244.05	1	< 0.001
Rural	2.6			
Education				
No education	6.12	5.40E+03	3	< 0.001
Primary	3.45			
Secondary	1.5			
Higher	0.6			
Employment				
Not employed	1.91	36.25	1	< 0.001
Employed	2.7			
Wealth index				
Poorest	2.86	413.08	4	< 0.001
Poorer	2.87			
Middle	2.45			
Richer	1.99			
Richest	1.12			
Caste				
SC	2.24	235.75	3	< 0.001
ST	1.99			
OBC	2.46			
Others	1.93			
Religion				
Hindu	2.31	170.82	2	< 0.001
Muslim	2			
Others	1.63			
Marital status				
Never married	0.43	4.20E+03	2	< 0.001
Married	3.37			
Widowed/divorced/Separated	4.09			
Region				
Northern	2.65	304.10	5	< 0.001
Western	2.63			
Southern	2.19			
Eastern	1.27			
Central	1.36			
North-Eastern	1.69			
Smoking				
No	2.22	24.54	1	< 0.001
Yes	4.05			
Drinking				
No	2.22	40.37	1	< 0.001
Yes	4.1			
Regular consumption of fried foods and aerated drinks				
No	2.12	36.04	1	< 0.001
Yes	2.27			
Age at Menarche				
12 or less	1.06	88.31	2	< 0.001
13–15	0.44			
More than 15	0.5			
Nulliparity				
No	3.76	4.90E+03	1	< 0.001
Yes	0.56			
Menstrual hygiene				
Hygienic methods	0.4	65.65	2	< 0.001
Unhygienic methods	0.76			
Both	0.4			
Age at first birth				
Below 18	6.25	1.20E+03	2	< 0.001
18–24	3.26			
25+	1.57			
Hormonal contraceptive use				
No	2.26	8.57	1	< 0.001
Yes	1.95			
Had terminated pregnancy				
No	2.08	416.78	1	< 0.001
Yes	3.53			
BMI category				
Underweight	1.64	563.24	3	< 0.001
Normal weight	2.11			
Overweight	3.21			
Obese	3.52			
Anaemic				
No	2.5	119.67	1	< 0.001
Yes	1.99			
Glucose level				
Non-diabetic	2.23	148.33	1	< 0.001
Diabetic	5.09			

Tobacco (4%), alcohol (4.1%) and regular fried food (2.3%) consumption are observed to be significantly associated with premature menopause. The women whose age at menarche is more than 15 years, have higher percentage of premature menopause (1.1%). Nulliparity was not observed to be a significant correlate. Premature menopause was comparatively higher among those who used unhygienic methods of menstruation (0.76%). Those whose age at first birth was below 18 years of age, premature menopause was higher among them (6.3%). There is also an observed significant association of premature menopause with hormonal contraceptive use (1.9%) and termination of pregnancy (3.5%). Overweight (3.2%) and obese (3.5%) women are observed to experience premature menopause in comparison to lower BMI categories. Premature menopause is significantly higher among diabetic women (5.1%) (Table 2).

For the sampled age-group 40–44 years for measuring early menopause, the bivariate analysis is displayed in Table 3. Menopause evidently increases with increasing age, and majority, around 23% attained at the age 44. The patterns are quite similar as premature menopause, where women residing in rural areas (18.1%), has no education (21%), belonging to poor wealth quintile (19%), and scheduled caste (17.4%), has significantly higher percentage of experiencing early menopause. Marital status also showed and significant association with early menopause, with around 20% prevalence among those who were widowed/divorced/separated. Western regions showed significantly higher proportion of early menopausal women (18.89%).

**Table 3:** Cross-tabulations and chi-square tests of early menopause with the possible correlates (age group 40–44).

	Early Menopause (%)	χ^2^ value	DF	*p*-value
Age				
40	12.65	819.38	4	< 0.001
41	12.83			
42	16.57			
43	18.69			
44	22.94			
Residence				
Urban	12.76	144.89	1	< 0.001
Rural	18.07			
Education				
No education	20.91	977.24	3	< 0.001
Primary	17.26			
Secondary	12.9			
Higher	6.29			
Employment				
Not employed	17.12	0.01	1	> 0.001
Employed	16.49			
Wealth index				
Poorest	18.89	298.02	4	< 0.001
Poorer	19.04			
Middle	17.98			
Richer	15.24			
Richest	11.14			
Caste				
SC	17.42	170.73	3	< 0.001
ST	15.93			
OBC	16.81			
Others	14.44			
Religion				
Hindu	16.36	185.85	2	< 0.001
Muslim	16.52			
Others	13.34			
Marital status				
Never married	12.39	113.04	2	< 0.001
Married	15.87			
Widowed/divorced/Separated	20.13			
Regions				
Northern	15.56	261.38	5	< 0.001
Western	18.89			
Southern	16.01			
Eastern	13.25			
Central	14.65			
North-Eastern	14.31			
Smoking				
No	16.1	5.16	1	< 0.05
Yes	21.71			
Drinking				
No	16.15	3.94	1	< 0.05
Yes	20.06			
Regular consumption of fried foods and aerated drinks				
No	15.79	14.85	1	< 0.001
Yes	16.35			
Nulliparity				
No	16.15	1.02	1	> 0.001
Yes	17.44			
Age at first birth				
Below 18	24.16	1.30E + 03	2	< 0.001
18–24	15.36			
25+	8.27			
Hormonal contraceptive use				
No	16.72	118.4	1	< 0.001
Yes	12.64			
Had terminated pregnancy				
No	16.06	9.84	1	< 0.001
Yes	16.91			
BMI category				
Underweight	20.44	125.93	3	< 0.001
Normal weight	15.89			
Overweight	15.92			
Obese	15.87			
Anemic				
No	18.29	124.42	1	< 0.001
Yes	14.38			
Glucose level				
Non-diabetic	16.05	31.94	1	< 0.001
Diabetic	21.31			

Consumption of tobacco (21.7%), alcohol (20.1%) and regular fried food (16.4%) are found to be significantly associated with early menopause. Early menopause is higher among those whose age at first birth was below 18 years of age (24.1%). There is also an observed significant association of early menopause with hormonal contraceptive use (12.6%) and termination of pregnancy (16.9%). On the contrary to premature menopause, early menopause is higher among underweight women (20.4%). Early menopause is significantly higher among diabetic women (21.3%) (Table 3).

*Results from Survival models:* The multiple cox-proportional hazard models taking premature and early menopause as the event is displayed in Table 4 and Table S1. The model 3 and 4, is observed to be predicting premature menopause as an event the best. The results are reported for the model 4, where all the possible explanatory variables are adjusted.

**Table 4:** Adjusted hazard ratios and its confidence intervals, for observing the effect of covariates on the occurrence of premature menopause.

	Model 1	Model 2	Model 3	Model 4
Residence				
Urban ®				
Rural	1.016 (0.875–1.179)	1.023 (0.881–1.188)	0.613 (0.398–1.042)	0.672 (0.427–1.060)
No education ®				
Primary	2.673 (0.689–10.37)	1.785 (0.425–7.494)	0.734*** (0.621–0.868)	0.733*** (0.620–0.867)
Secondary	3.103* (0.960–10.03)	2.673 (0.822–8.690)	0.676*** (0.589–0.775)	0.672*** (0.586–0.770)
Higher	1.580 (0.445–5.618)	1.408 (0.391–5.073)	0.413*** (0.313–0.545)	0.410*** (0.310–0.541)
Employment				
Not employed ®				
Employed	0.879 (0.780–1.190)	0.878 (0.779–1.189)	1.188** (1.073–1.371)	1.136*** (1.032–1.217)
Wealth index				
Poorest ®				
Poorer	1.134 (0.964–1.335)	1.134 (0.964–1.335)	0.832 (0.476–1.454)	0.917 (0.513–1.641)
Middle	1.100 (0.926–1.305)	1.102 (0.928–1.309)	0.985 (0.559–1.736)	1.093 (0.602–1.984)
Richer	0.914 (0.750–1.113)	0.919 (0.753–1.121)	0.844 (0.450–1.583)	0.865 (0.441–1.694)
Richest	0.579 (0.268–1.253)	0.716 (0.322–1.592)	0.623*** (0.486–0.798)	0.632*** (0.492–0.812)
Caste				
SC ®				
ST	0.997 (0.825–1.205)	0.986 (0.814–1.193)	0.748 (0.418–1.341)	0.681 (0.371–1.247)
OBC	0.847 (0.685–1.046)	0.839 (0.677–1.040)	0.671 (0.350–1.288)	0.615 (0.313–1.209)
Others	0.873 (0.546–1.395)	0.837 (0.514–1.365)	0.805 (0.520–1.521)	0.802 (1.117–1.517)
Religion				
Hindu ®				
Muslim	0.934 (0.781–1.117)	0.911 (0.758–1.096)	1.233 (0.737–2.063)	1.188 (0.683–2.068)
Others	0.841 (0.678–1.043)	0.838 (0.676–1.040)	1.465 (0.818–2.626)	1.719* (0.955–3.095)
Marital status				
Never married ®				
Married	0.999 (0.873–1.185)	0.986 (0.814–1.193)	1.012 (0.978–1.334)	1.347* (1.126–1.762)
Widowed/divorced/separated	0.893 (0.686–1.248)	0.839 (0.677–1.040)	2.134** (1.965–2.335)	2.671** (1.890–2.793)
Regions				
North ®				
West	1.183*** (1.077–1.317)	1.238*** (1.091–1.455)	1.259*** (1.087–1.459)	1.257*** (1.083–1.459)
South	1.221*** (1.078–1.389)	1.258*** (1.097–1.487)	1.261*** (1.086–1.464)	1.262*** (1.086–1.466)
East	0.729 (0.478–1.306)	0.893 (0.589–1.158)	0.822 (0.584–1.256)	0.820 (0.583–1.154)
Central	1.287** (1.103–1.692)	1.322** (1.084–1.712)	1.326** (1.034–1.700)	1.327** (1.034–1.701)
North-east	1.159 (0.967–1.273)	1.152 (0.964–1.226)	1.145 (0.988–1.325)	1.142 (0.985–1.323)
Smoking				
No ®				
Yes		0.795 (0.509–1.241)	1.340** (1.117–1.529)	1.208** (1.123–1.490)
Drinking				
No ®				
Yes		1.015 (0.716–1.439)	1.894 (0.452–7.933)	1.004 (0.136–7.394)
Regular consumption of fried foods and aerated drinks				
No ®				
Yes		1.072** (1.037–1.227)	1.435** (1.086–2.323)	1.443* (1.180–2.367)
Age at Menarche				
﻿More than 15s ®				
﻿12 or less			3.190*** (1.696–6.000)	2.630*** (1.365–5.071)
﻿13–15			0.974 (0.610–1.555)﻿	0.859 (0.535–1.380)
Nulliparity				
No ®				
Yes			0.914 (0.441–1.892)	0.875 (0.404–1.894)
Menstrual hygiene				
Hygienic methods ®				
Unhygienic methods			1.660** (1.025–2.687)	1.579* (1.056–2.608)
Both			1.399 (0.914–2.143)	1.264 (0.809–1.974)
Age at first birth				
Below 18 ®				
18–24			0.381 (0.136–1.062)	0.318* (0.106–0.954)
25+			0.887 (0.457–1.722)	0.887 (0.439–1.792)
Hormonal contraceptive use				
No ®				
Yes			1.472* (1.142–1.573)	1.535** (1.159–1.795)
Had terminated pregnancy				
No ®				
Yes			0.896 (0.313–2.561)	1.028* (1.013–1.941)
BMI category				
Underweight ®				
Normal weight				0.545*** (0.368–0.805)
Overweight				0.303** (0.107–0.854)
Obese				0.866 (0.258–2.905)
Anemic				
No ®				
Yes				0.927 (0.640–1.343)
Glucose level				
Non-diabetic ®				
Diabetic				3.579** (3.473–4.07)

®Reference category.

***p < 0.001; **p < 0.01; *p < 0.05.

The survival analysis demonstrated that women with higher levels of education have lower chances of having premature menopause, in comparison with those who have no education. Women who are employed have a 13.6% higher probability of experiencing premature menopause in comparison to unemployed women (HR 1.136; *p* < 0.001). The women in higher wealth quintile, that is, richest have 36.8% lower chances of having premature menopause than poorest women (HR 0.632; *p* < 0.001). Widowed/divorced/separated women are at a much higher risk of premature menopause in comparison to never married (HR 2.671; *p* < 0.01). Region as a control showed that in the central and southern region of the country the risk of experiencing premature menopause is 32.7% (HR 1.327; *p* < 0.05) and 26.2% (HR 1.262; *p* < 0.01) higher respectively, in comparison to Northern regions.

Smoking is observed to be a significant predictor of premature menopause, with 20.8% higher risk (HR 1.208; *p* < 0.01). Regular consumption of fried food increases the risk off experiencing premature menopause by 44.3% (HR 1.443; *p* < 0.05). There is increased risk of premature menopause for the one whose age at menarche is 12 or less years compare to 15 or more age at menarche (HR 2.63; *p* < 0.001). Practicing unhygienic menstrual methods increases the risk by 57.9% in comparison to those who practice hygienic methods (HR 1.579; *p* < 0.05). There is lower risk of premature menopause for those the women whose age at first birth is 18–24 in comparison to if the age at first birth is below 18 years (HR 0.318; *p* < 0.05). The risk was also higher for women who had ever used any of the hormonal contraceptives (HR 1.535; *p* < 0.05). The women who ever had any terminated pregnancy have higher changes of attaining menopause prematurely (HR 1.028; *p* < 0.05). The diabetic women have 3.5 times higher chances of experiencing premature menopause (HR 3.579; *p* < 0.01) (Table 4). The result for early menopause followed a similar pattern with smoking (HR 1.134; *p* < 0.001) and termination of pregnancy (HR 1.116; *p* < 0.001) being two of the significant predictors (Table S1).

## Discussion

The study estimated the prevalence of premature and early menopause, and further analyzed its determining factors, utilizing a large-scale national population survey. Previous studies based on NFHS or other national surveys mostly included women who underwent surgical menopause as well^31–33^, however this study excluded it in order to avoid overestimation of the prevalence rates for premature and early menopause, with the confounding effect of large number of hysterectomies. The estimated prevalence of premature menopause is 2.2% and early menopause is 16.2%. A study by^34^, had estimated premature menopause to be 1.5% using the DLHS 2007–2008 data in India. Another study based on the US and Korean population too computed the prevalence of premature menopause, as 1.7% and 2.8%, respectively, and early menopause as 3.4% and 7.2% in US and Korea, respectively^35^. However, none of the studies had excluded the pregnant and lactating mothers from the sample, which was considered in the present study, thus making the current estimation more robust.

Overall, the study's socioeconomic, family planning, and demographic characteristics were substantially linked to premature and early menopause. Women in rural settings are more likely than those in urban areas to have premature menopause because they are less likely to have access to health care services^36^. The degree of education has been demonstrated to be a significant explanatory factor in premature and early menopause. Premature and early menopause rates increased among women with lower levels of education, This result is in line with other studies as well^16,37^. According to the present study, women from poorer households were more likely to go through a premature and early menopause, which is consistent with earlier studies in India or other regions^15,18^. There is a possible poverty and nutrition link in this regard. This phenomenon could be explained in the way that women in rural areas in poorer households, and also having less or no education have lack of awareness coupled with inaccessibility of healthcare services and poor nutritional diet. The intersectionality of residential, economic and educational vulnerability has compounded effect that may lead to early onset of menopause.

Lifestyle choices such as tobacco, alcohol and junk food consumption were observed to be contributing factors towards premature and early menopause. Tobacco smoking during reproductive cycle, or in general smoking had been found to be a significant factor causing premature menopause^32^. The risk was found to be comparatively higher among female cigarette smokers than the women who did quit smoking. By lowering the flow of oestrogen, tobacco's anti-estrogen actions speed up the start of menopause^38^. Overall, this compromises the typical hormonal balance in women, which has an impact on the entire system. When smoking combined with an early menopause, the hormonal imbalances result in sleep problems, depressive symptoms, and eventually impaired cognitive performance^20,39,40^.

Meat and alcohol consumption and physical inactivity, were independently found to have significant association with premature menopause in previous studies^41,42^. Dietary pattern and nutritional status of a woman had been also found to be contributing factors to premature menopause. This risk is higher among women with low body mass index (BMI) or malnourished which is also observed from the study results^43,44^. Fat tissue is where oestrogen is stored, and extremely thin women have lower reserves of oestrogen, which are more easily exhausted. Women with higher BMIs have higher amounts of estrone (E1) and estradiol (E2) in their bodies, which can delay menopause. BMI is a key factor in determining endogenous oestrogen levels^45^. Though, they study showed premature menopause to be higher among overweight/obese women, whereas early menopause was higher among underweight women.

Early age at menarche if found to have a significant association with premature and early menopause. A study by Mishra et al. (2017), reported that women who had menarche before age of 13, had double the risk of experiencing premature menopause and 31% higher risk of early menopause^46^. Early menarche has been linked to poor reproductive functioning, including irregular periods^47,48^, PCOS^49^, and a slightly higher risk of endometriosis^50^, according to earlier studies. In contrary to previous studies^46,48^ that reported the nulliparous women had higher chances of experiencing early onset of menopause, the present study did not show any significant association. The age at first birth is another attributable factor, which shows that premature and early menopause is higher among the women who age at first birth is lower than 18 years^33^. Women who do not become pregnant typically have an earlier menopause than those who have children. Additionally, it is true that common factors—ranging from genetics to childhood environmental factors like obesity, psychological stress, and social environment—may explain the association and have an impact on menopause, the date of the first period, and fertility^46^.

Interestingly the use of hormonal contraceptives such as, injectables, pills, and emergency contraception also emerged as an explanatory variable for premature and early menopause. Though there has been studies earlier that stated those who used oral contraception had experienced premature menopause less^51–53^. History of termination of pregnancy showed association with premature and early menopause, however not established much in earlier studies. In addition to harming the uterus, abortion can result in ovarian dysfunction, which can lead to the failure of the ovaries. Able to produce eggs of average, everyday quality. Hormone production volume can also be impacted. Additionally, ovarian infection or blocked fallopian tubes may result from unsafe or ongoing abortion, full ovary removal, which harms and ages the ovaries^54^.

Another interesting result is that of the association of higher glucose level with premature and early menopause. Women who have either type 1 diabetes (before age 30) or type 2 diabetes (between 30 and 39 years) are more likely than identical women without diabetes to experience menopause earlier in life. There might be a connection between diabetes and changes in the body, reproductive system, and ageing and functioning of the ovaries^55^. Future studies are required to determine the potential causes of the association between diabetes and premature or early menopause. The study provides a baseline for carrying out future research in India by considering more factors and conducting causal inferences.

## Strengths and limitations

The study has several strengths owing to its national representativeness and methodological robustness. Since the analysis is based on a national-level population survey that covered large-scale data on women’s health that helped to assess the maximum factors affecting early age at menopause. It was also possible to exclude the women who had undergone hysterectomy as with the surgery done these women have lower estrogen levels than other women who have attained premature or early menopause naturally^56^. This analysis controlled for most of the factors that could confound the results.

The study has some limitations owing to the cross-sectional nature. There are chances of recall bias of the date of last menstrual cycle since it is self-reported. The fact that the current study employed data from women between the ages of 15 and 49 was a clear restriction. In order to get over this restriction, we employed survival models in our research. These models work best with data that is 'time to event data' and that contains censored cases. Since the study has analysed secondary data, more detailed micro-studies would help in better understanding of the premature or early menopausal cases.

## Conclusion

The high number of women attaining menopause at younger ages is a matter of concern. This study is an important contribution to literature that provides robust prevalence estimate for premature and early menopause, and holistically analyses possible explanatory factors using a large-scale national data. It is crucial that underprivileged women have access to the proper diet and healthcare measures because early menopause is associated with osteoporosis and other health problems. To further understand the relationships between general undernutrition brought on by poverty and specific micronutrient deficiencies that may have an impact on ovarian reserve, such as vitamin D deficiency, more research is required. In India, both the proportion and the absolute number of post-menopausal women are growing, therefore it's critical to revamp public reproductive healthcare facilities to include the right kinds of treatment for them.

The current health care system faces a difficulty in providing adequate care for the many women who experience early menopause. The demands of women going through early menopause should be taken into account by the government programmes already in place that are designed to meet the needs of childbearing women. Women who are approaching menopause want assistance in managing the symptoms brought on by hormonal shift and the menopausal transition. Women may endure vasomotor symptoms, urinogenital issues, and psychological issues during this time. These women may be able to cope with the discomfort they experience with the support of the therapy and counselling that health care professionals can offer. It is possible to teach healthcare professionals to offer the required counselling and advice to deal with the issues faced by menopausal women; these actions are low-cost and simple to incorporate into current programmes. Another strategy to encourage women going through premature menopause to seek out the appropriate medical care is to raise public knowledge of the detrimental effects of premature menopause on health and the significance of doing so. It is suggested to ensure health care access to underprivileged women has been supported by the data in wealth index, social and demographic characteristics, and lifestyle habits, and there is dire need to expand health care services and thus the budgetary allocations.

### Supplementary Information

Supplementary Information.

## Data Availability

Data was requested and obtained from concerned authorities. The data is freely available in the DHS website on request (https://dhsprogram.com/data/available-datasets.cfm).
