# Supplementary material for: Exploring the triggers of premature and early menopause in India: a comprehensive analysis based on National Family Health Survey, 2019–2021
Source: Sci Rep. 2024 Feb 6;14:3040. doi: 10.1038/s41598-024-53536-9 (PMC10847426; doi:10.1038/s41598-024-53536-9)
Supplement: Supplementary file 1 — Supplementary Information. [file 41598_2024_53536_MOESM1_ESM.docx]

**Table S1:** Adjusted hazard ratios and it confidence intervals, for association of covariates with early menopause

|  |  | Model 1 | Model 2 | Model 3 | Model 4 |
| --- | --- | --- | --- | --- | --- |
| Residence | Urban ® |  |  |  |  |
|  | Rural | 1.011(0.892 - 1.146) | 1.015(0.894 - 1.151) | 1.022(0.901 - 1.160) | 1.032(0.904 - 1.177) |
| Education | No education ® |  |  |  |  |
|  | Primary | 0.771***(0.674 - 0.881) | 0.770***(0.673 - 0.880) | 0.781***(0.683 - 0.894) | 0.796***(0.694 - 0.914) |
|  | Secondary | 0.658***(0.585 - 0.740) | 0.657***(0.585 - 0.739) | 0.698***(0.620 - 0.787) | 0.719***(0.636 - 0.812) |
|  | Higher | 0.349***(0.258 - 0.472) | 0.349***(0.258 - 0.473) | 0.381***(0.281 - 0.517) | 0.407***(0.299 - 0.555) |
| Employment | Not employed ® |  |  |  |  |
|  | Employed | 1.010(0.917 - 1.113) | 1.009(0.916 - 1.111) | 1.132**(1.101 - 1.301) | 1.142**(1.093 - 1.896) |
| Wealth index | Poorest ® |  |  |  |  |
|  | Poorer | 0.991(0.863 - 1.138) | 0.992(0.864 - 1.140) | 0.991(0.863 - 1.139) | 0.994(0.862 - 1.145) |
|  | Middle | 0.965(0.834 - 1.117) | 0.965(0.834 - 1.118) | 0.954(0.824 - 1.105) | 0.970(0.834 - 1.128) |
|  | Richer | 0.927(0.789 - 1.090) | 0.931(0.791 - 1.095) | 0.925(0.786 - 1.088) | 0.913(0.770 - 1.082) |
|  | Richest | 0.850(0.699 - 1.033) | 0.856(0.702 - 1.044) | 0.856(0.702 - 1.044) | 0.878(0.712 - 1.081) |
| Caste | SC ® |  |  |  |  |
|  | ST | 1.046(0.901 - 1.214) | 1.041(0.896 - 1.210) | 1.036(0.891 - 1.205) | 1.014(0.868 - 1.184) |
|  | OBC | 0.780***(0.655 - 0.930) | 0.777***(0.650 - 0.928) | 0.785***(0.656 - 0.938) | 0.779***(0.649 - 0.934) |
|  | Others | 1.075(0.949 - 1.217) | 1.073(0.948 - 1.215) | 1.063(0.939 - 1.204) | 1.047(0.922 - 1.190) |
| Religion | Hindu ® |  |  |  |  |
|  | Muslim | 1.027(0.881 - 1.197) | 1.020(0.870 - 1.195) | 1.047(0.893 - 1.227) | 1.047(0.889 - 1.234) |
|  | Others | 0.780***(0.654 - 0.930) | 0.778***(0.652 - 0.927) | 0.799**(0.669 - 0.953) | 0.760***(0.632 - 0.914) |
| Marital status | Never married ® |  |  |  |  |
|  | Married | 0.692(0.444 - 1.077) | 0.688(0.531 - 1.335) | 0.877(0.645 - 1.027) | 0.883(0.634 - 1.108) |
|  | Widowed/divorced/separated | 0.842(0.531 - 1.334) | 0.838(0.529 - 1.329) | 0.999(0.723 - 1.113) | 1.067**(1.013 - 1.128) |
| Regions | North ® |  |  |  |  |
|  | West | 1.397***(1.318 - 1.481) | 1.333***(1.257 - 1.414) | 1.322***(1.244 - 1.404) | 1.281***(1.204 - 1.362) |
|  | South | 1.277***(1.202 - 1.357) | 1.275***(1.199 - 1.356) | 1.279***(1.201 - 1.363) | 1.291***(1.211 - 1.376) |
|  | East | 0.62***(0.54 - 0.712) | 0.658***(0.572 - 0.756) | 0.656***(0.569 - 0.755) | 0.649***(0.563 - 0.748) |
|  | Central | 1.102*(0.994 - 1.221) | 1.104*(0.994 - 1.226) | 1.178***(1.059 - 1.311) | 1.203***(1.08 - 1.341) |
|  | North-east | 1.098***(1.035 - 1.166) | 1.063**(1.001 - 1.13) | 1.05(0.987 - 1.117) | 1.042(0.979 - 1.11) |
| Smoking | No ® |  |  |  |  |
|  | Yes |  | 1.117(0.852 - 1.464) | 1.107**(1.045 - 1.452) | 1.134***(1.064 - 1.487) |
| Drinking | No ® |  |  |  |  |
|  | Yes |  | 0.971(0.732 - 1.288) | 0.980(0.738 - 1.300) | 1.000(0.749 - 1.334) |
| Regular consumption of fried foods and aerated drinks | No ® |  |  |  |  |
|  | Yes |  | 1.019(0.913 - 1.138) | 1.028(0.921 - 1.148) | 1.053(0.940 - 1.179) |
| Nulliparity | No ® |  |  |  |  |
|  | Yes |  |  | 0.992(0.766 - 1.285) | 0.926(0.702 - 1.223) |
| Age at first birth | below 18 ® |  |  |  |  |
|  | 18-24 |  |  | 0.854(0.568 - 1.286) | 0.818(0.535 - 1.251) |
|  | 25+ |  |  | 0.679(0.455 - 1.015) | 0.634(0.418 - 1.064) |
| Hormonal contraceptive use | No ® |  |  |  |  |
|  | Yes |  |  | 0.812***(0.700 - 0.942) | 0.817***(0.702 - 0.952) |
| Had terminated pregnancy | No ® |  |  |  |  |
|  | Yes |  |  | 1.147**(1.014 - 1.298) | 1.116***(1.074 - 1.257) |
| BMI category | Underweight ® |  |  |  |  |
|  | Normal weight |  |  |  | 0.812***(0.701 - 0.941) |
|  | Overweight |  |  |  | 0.724***(0.610 - 0.859) |
|  | Obese |  |  |  | 0.861(0.692 - 1.071) |
| Anemic | No ® |  |  |  |  |
|  | Yes |  |  |  | 0.784***(0.713 - 0.861) |
| Glucose level | Non-diabetic ® |  |  |  |  |
|  | Diabetic |  |  |  | 0.971(0.743 - 1.268) |
| *Note: ® Reference category; *** p<0.001; ** p<0.01; * p<0.05* | | |  |  |  |
